# Feasibility and usability study of the ERES: A mobile application to intervene in emotional regulation and self-efficacy in minors with type 1 diabetes

**DOI:** 10.1177/20552076251348021

**Published:** 2025-06-20

**Authors:** Sofía García-Roldán, Sebastián Rubio, Sebastián Vivas, Rosario Castillo-Mayén, Carmen Tabernero, Concepción Muñoz, Bárbara Luque

**Affiliations:** 1 Department of Psychology, 16735 University of Cordoba, Córdoba, Spain; 2 215147 Maimonides Biomedical Research Institute of Cordoba (IMIBIC), Córdoba, Spain; 3 16501 Reina Sofia University Hospital, Córdoba, Spain; 4 Department of Specific Didactics, 16735 University of Cordoba, Córdoba, Spain; 5 Department of Psychology, 16779 University of Salamanca, Salamanca, Spain; 6 427640 Institute of Neurosciences of Castilla y León (INCYL), Salamanca, Spain

**Keywords:** Type 1 diabetes, eHealth, minors, usability, emotional regulation, self-efficacy

## Abstract

**Background:**

Type 1 diabetes (T1D) is one of the most prevalent endocrine diseases in childhood. The behavioral component is crucial for effective self-regulation of the disease. Psychological therapy improves emotional management, reducing some of the associated symptoms.

**Objective:**

The aim of this study is to analyze the use of a mobile application (app) (ERES) designed for training in emotional regulation and self-efficacy for children and adolescents with T1D.

**Methods:**

In mixed groups of eight participants, three face-to-face and five online sessions were combined in an 8-week intervention in a specific sequence. Users had the ERES app installed on their devices (mobiles or tablets), where they accessed different materials and activities to conduct online sessions.

**Results:**

A total of 28 patients, diagnosed with T1D, between 7 and 18 years old, started the intervention program, of whom 19 (*Mean_age_* = 11.63; *SD_age_* = 2.59; 12 girls) made use of the app (67.86%). To determine the level of usability of the app, the System Usability Scale (SUS) was applied, whose results were favorable. We found an average score of 71.1 for usability and 75.66 for learnability, both within a good range of satisfaction. The overall mean scale score was 72.5, which consists of a participants’ good adherence to treatment.

**Conclusions:**

This is one of the first studies to analyze and develop the feasibility and usability of an app to improve emotional well-being in minors with T1D. The results show that ERES is a feasible tool with good levels of usability and ease of learning, which allows the improvement of patients’ well-being.

## Introduction

The International Diabetes Federation (IDF) (2022) reported that, in 2022, there were 8.75 million individuals living with type 1 diabetes (T1D). Among this total, 1.52 million (17.0%) were under the age of 20.^1^ More than 17,000 minors in Spain had T1D in 2021. During the last few years, there has been a constant increase in the rates of T1D in minors.^2^ Diabetes care and management needs directly affect children and adolescents, which has led to the need for more specialized interventions and care to aid in the management of diabetes in minors and parents, who play an important role in the management of their children’s disease.^3^ T1D is difficult to manage in this population due to constant demands for control, which increases discomfort about diabetes and fear of poor glucose control.^4^ Research revealed that adolescents with T1D are at a significant risk for psychiatric disorders (10–20%), eating disorders (8–30%), and substance abuse (25–50%), which often lead to treatment non-compliance and worsening metabolic control.^5^ In addition, adolescents with T1D are at a heightened risk of depression compared to their peers. This increased vulnerability is attributed to the added pressures of managing their condition, the effort to gain independence from their parents, and the challenges associated with exploring and defining their identity.^6^ On the other hand, family stress in these cases could hinder family dynamics, affecting children’s well-being.^7^ Children with diabetes have been seen to be more likely to experience psychological difficulties^8^; for example, in the study of Helgeson et al.,^9^ it was seen that adolescents present difficulties in social acceptance compared to healthy adolescents. In addition, girls show decreased self-esteem. It has also been revealed that in young people with T1D are prevalent anxiety symptoms, whose levels are associated with higher glycosylated hemoglobin (HbA1c) levels, worse self-management and coping behaviors, depressive symptoms, fear of hypoglycemia, and lower frequency of blood glucose monitoring.^4^ In addition, depressive symptoms are associated with poor disease self-management and poor glycemic control.^10,11^

The increasingly common use of technology for the management of diabetes in children has been shown to be beneficial both for the psychosocial functioning of parents, and consequently the well-being of children, and for the improvement of glycemic levels.^12^ Some applications have been designed to help glycemic control such as OneTouch Reveal®, SocialDiabetes, mySugr: diabetes diary app®, Diabetes menu®, Tactio SALUD®, and Diabetes:M®, which have obtained the best quality and usability indexes according to the review by Quevedo-Rodríguez and Wägner.^13^ However, other mobile applications have had a more educational function, such as *T1D*, designed for minors aged 11–16 years with T1D, which has the main objective of helping to understand the disease to improve adherence to treatment,^14^ and MyDiabetic, which seeks to educate children about the relationship between diet, insulin, and physical activity and provide knowledge on the use of glucometers, insulin pens, and advanced technologies for diabetes management, such as glucagon, ketoacidosis detection, and continuous glucose monitoring.^15^

Emotional regulation is the ability to identify, understand, and manage one’s own emotions, considered an adaptive skill.^16,17^ Adolescence is identified as a critical stage for the development of this skill, with significant implications for mental health.^18^ This stage influenced by the presence of a chronic disease increases the need to acquire good emotional regulation skills. On the one hand, emotions suppose a relevant impact on physical health, and on the other hand, the appearance of some emotions could interfere with treatment. The literature indicates that the most frequent psychological disorders among people with diabetes are anxiety and depression.^19^ Consequently, treatment of some psychological variables such as anxiety symptoms is also associated with improved glycemic control.^20^ Psychological factors such as depression or diabetes stress are largely related to glycemic control, as it is a stress caused by the demands of diabetes control.^21^ Therefore, in recent years, in parallel with the empowerment of mental health, the focus has been placed on studying the psychosocial variables that influence T1D care.^22^ With regard to the design of interventions aimed at children and adolescents with T1D, psychoeducational interventions have shown great potential to improve disease management and other psychosocial variables,^23^ obtaining very favorable results by improving HbA1c levels after their application^24,25^ even in online format.^26^ As for predominantly psychological interventions aimed at increasing adherence, improving metabolic control, and coping with stress in adolescents, different techniques have been developed, such as mindfulness, which promotes self-care in pediatric patients with T1D,^19^ and those based on cognitive-behavioral therapy (CBT) and training in coping and stress management skills.^27^ It could be considered that the need to manage emotions primarily involves adolescents, who, in their pursuit of independence, may sometimes rebel against self-care behaviors, thereby deteriorating treatment adherence.^28^ However, emotional support plays a very important role for younger children and their families, who experience an invasive onset process and must adapt to a new lifestyle, in addition to managing decision-making and uncertainty about the future.^29^ In this case, young children must cope with the significant impact on their family and social life.^30^

According to Bandura’s self-efficacy theory, one’s beliefs about their ability to handle certain situations influence the coping strategies they use. These self-efficacy beliefs determine the level of effort exerted to achieve a goal.^31,32^ Since managing diabetes requires specific daily actions, high self-efficacy is associated with better disease management.^33^ Tabernero et al. studied the relevance of self-efficacy beliefs in controlling diabetes and predicting behaviors and health promotion in children and adolescents with T1D.^34^ The results were reflected in biomedical indicators such as glycemic levels. It was found that adolescents with high self-efficacy maintained correct blood sugar levels for longer periods. However, this relationship was not observed in children under 14 years of age. This age-based distinction arose because younger children’s T1D management is typically carried out by their parents,^34^ whereas adolescents take control themselves.^35^ Nevertheless, adolescents had poorer glycemic indices than younger children.^34^ These findings clearly justify the need to promote self-efficacy both during childhood—to gradually acquire responsibility for T1D management—and during adolescence to achieve better adherence to treatment.

By combining new technologies with the consideration of psychological variables in the population with T1D, different proposals for eHealth applications have been designed, mostly for adults.^36^ For about a decade, new studies have been carried out with the aim of creating a good model of online psychoeducational intervention for children with T1D. At the beginning of the second decade of the 21st century, the results of a coping skills training program for this same population (TEENCOPE) were published, in which greater coping and stress reduction were achieved among participants.^37^ Subsequently, the effectiveness of another program was studied, this time of a psychoeducational approach (Teens.Connect), and despite low participation, a decrease in perceived stress was achieved over time.^26^ Currently, progress continues to be made in the creation of new online interventions from different perspectives to respond to the variety of psychological needs presented among the population of children and adolescents with T1D, such as Diabetes Journey and THR1VE among the most recent ones.^38,39^

Our intervention is one of the first with a semi-face-to-face format. The face-to-face part is preserved because it is a way for these patients to support each other to improve self-care and improve adherence to treatment, while professionals can receive information about their needs by learning about their experiences first-hand.^40,41^ The online part will be carried out with the ERES (Healthy Emotional Regulation Training) app, designed for this purpose. Through the intervention, the aim is to increase self-efficacy and to acquire emotional regulation strategies for better coping with the disease.

According to the American Psychological Association,^42^ minors with chronic illnesses receive treatment that involves psychological and behavioral components. These interventions are divided into five categories: educational (focused on disease knowledge and skill acquisition), behavioral (targeting specific issues, such as maladaptive behaviors or barriers to treatment adherence), family-based (essential for managing the disease in younger patients), psychological (centered on emotional well-being to improve mental health while adapting to living with and managing the disease), and digital health interventions (dedicated to enhancing healthy behaviors and disease management through electronic devices).^43^

Specifically, in patients with T1D, psychological interventions mainly include training in coping skills, social problem-solving, communication skills, conflict resolution, and more.^37^ Both coping skills training and CBT appear to benefit youths’ psychological symptoms as well as their self-management behaviors and glycemic control.^44–47^ These types of interventions are typically conducted in person. However, digital health interventions are delivered via electronic devices, often mobile devices, aiming to improve adherence to glycemic control through continuous glucose monitoring and reminders.^43^ Evidence suggests that such interventions tend to work better in adults with T1D.^48^

To achieve this level of treatment adherence in children and adolescents, greater self-management of the disease should be promoted from a psychoeducational perspective. In the systematic review by Luque et al.^23^ on psychoeducational interventions in recent years for children and adolescents, it is concluded that while optimal blood glucose levels are relevant outcomes, it is also important to consider other psychosocial variables that contribute to better disease control, such as self-management. In this review, effectiveness indicators included that the intervention design aligns with the setting and population, that educator psychologists are involved in the design and supervision, and that resources and educational methods, especially the use of digital tools and peer interaction strategies, are chosen correctly. Both aspects appear to have the potential to positively influence diabetes education and, as a result, improve disease management.^23^ In conclusion, following the recommendations of previous studies, we aim to design an intervention that meets current needs, as despite the variety of existing interventions, it remains important to continue researching theoretical approaches and applicable methods, since education can improve diabetes control.^49–51^

### Related works

Mobile applications for the management of pediatric chronic diseases have been evaluated in terms of feasibility and usability, considering factors such as adherence and effectiveness in disease self-management. iBDecide, designed for adolescents with inflammatory bowel diseases, aims to facilitate medical decision-making through interactive information. Its evaluation focused on information gathering and usability testing using the System Usability Scale (SUS).^52,53^ Similarly, the study on the Asthma Control Test (ACT) aimed to examine the feasibility and utilization of a mobile asthma action plan (AAP) among adolescents, allowing them to assess their disease control. To evaluate feasibility, the study assessed application usage frequency, patient satisfaction, and the impact of its use on asthma self-efficacy and asthma control over an 8-week period.^54^ Roadmap 1.0, in turn, promoted health education and self-management in adolescents with chronic diseases through interactive modules, measuring adoption and user engagement variables such as login frequency, days of access, total minutes of utilization, and overall usage.^55^ YouthCOACHCD, designed for young individuals with Crohn’s disease, focused on improving treatment adherence through CBT-based strategies, evaluating its effectiveness and acceptance through adherence and dropout rates, formative user feedback, potential side effects, intervention satisfaction, and therapeutic alliance.^56^

In the context of T1D, several applications have prioritized glucose monitoring as the core component of intervention. Bant enabled adolescents to log glucose levels and share data with healthcare professionals, incorporating gamification to enhance adherence.^57^ Glucophone™, on the other hand, integrated continuous monitoring with alerts and real-time feedback to improve disease management.^58^ Finally, Diamob and the Diabetes Message System facilitated doctor–patient communication, promoting continuous data logging and evaluating interaction as a key feasibility indicator, along with app usage frequency (daily blood glucose readings) and satisfaction.^59^

Other applications have approached T1D management from a psychoeducational perspective. MyT1DHero combined education and social support through an interactive and gamified interface, evaluating adoption rates, app usage, intervention satisfaction, and usability through a questionnaire.^60^ MedVenture, focused on gamification, introduced challenges and missions to encourage healthy habits, measuring both usage and usability.^61^ Lastly, the Healthcare Chief Executive Officer (CEO) app, designed to support the self-management of adolescents with T1D during their transition to early adulthood, determined its feasibility through heuristic evaluation to identify usability issues in the application interface, as well as think-aloud evaluation and a user satisfaction questionnaire.^62^

These studies highlight different approaches to the implementation of mobile applications for pediatric chronic diseases. Their feasibility assessments have relied on metrics such as adoption, usage, and satisfaction, providing valuable insights for the development of future digital interventions aimed at optimizing self-management and improving the quality of life of pediatric patients.

Unlike other applications that primarily focus on glucose monitoring (such as Bant, Glucophone™, Diamob, and the Diabetes Message System) or general education for disease self-management (such as Roadmap 1.0, iBDecide, and YouthCOACHCD), ERES integrates a psychoeducational approach based on CBT strategies, specifically designed for children and adolescents with T1D. While previous psychoeducational applications, such as MyT1DHero and MedVenture, have emphasized gamification and social support, ERES prioritizes the promotion of emotional regulation and self-efficacy to enhance disease self-management and psychological well-being.

This feasibility study assesses adoption, usage, and attrition rates by tracking the number of times participants access the application, providing an objective measure of engagement. This methodological approach contrasts with studies that primarily rely on self-perceived adherence or satisfaction as feasibility indicators. Furthermore, in alignment with previous research that has employed usability assessments (such as iBDecide and Healthcare CEO), the SUS has been utilized to evaluate user experience. By integrating these elements, ERES provides a more comprehensive evaluation framework that not only measures adherence metrics but also ensures feasibility and usability, offering valuable insights for the development of digital interventions aimed at managing T1D in pediatric populations.

The main contribution of this study, in line with previous research on treatments for patients with T1D, is the development of an effective intervention aimed at facilitating disease management in this population. To enhance treatment adherence—a common challenge among children and adolescents—we seek to assess the feasibility of a psychoeducational intervention supported by a mobile application. To this end, we analyze key indicators such as the number of participants who initiate, continue, and drop out of the intervention, which could serve as a preliminary trial for the main study. Additionally, we explore the potential to refine the intervention based on the results of the usability scale, whose scores across different items provide an indirect measure of participants’ satisfaction with the program.

### Study aim

The main objective of this study was to evaluate the feasibility and usability of an application in a sample of children and adolescents with T1D, as well as to support the intervention. Research conducted prior to a primary study is known as a feasibility study. They are employed to estimate crucial parameters required for the main study’s design. Importantly, the main research is responsible for evaluating the desired outcome; feasibility studies do not do so.^63^ Moreover, one crucial component of high-quality software is usability. Both user acceptance and well-being can be enhanced by improved usability. Thus, it has been acknowledged that usability is a crucial component of digital health technologies’ success.^64^ Thus, in an application of this nature, the goal is for it to meet both criteria.

## Methods

### Recruitment

A total sample of 37 participants were recruited from the endocrinology unit of the Reina Sofía University Hospital of Córdoba, Spain. Inclusion criteria were as follows: boys and girls aged 6–18 years with a confirmed diagnosis of T1D, who met the requirements for follow-up by the endocrinology consultation, had a smartphone or tablet at home with Internet access, and were not participating in other clinical trials or receiving psychological treatment.

A total of 28 participants started the intervention, of whom 19 made use of the app. The sample size is unusual for feasibility studies that do not assess the outcome of interest, so we relied on previous studies focused on eHealth interventions with minors with T1D, in which the number of participants ranged from 20 to 40.^57–60,62^

### Intervention

Participants received an intervention in emotional regulation and self-efficacy based on the Unified Protocol for the Transdiagnostic Treatment of Emotional Disorders in Children and Adolescents.^65^ The intervention consisted of a face-to-face group session + access to the ERES mobile app that has been registered in Clinical Trials (NCT06450730). The face-to-face emotional regulation intervention consisted of three sessions given by a clinical psychologist with previous training to groups of six to eight participants. The different sessions included the main components of the unified protocol for the transdiagnostic treatment of emotional disorders in children and adolescents adapted to the age (development of motivation for change, psychoeducation of emotions, the avoidance cycle, emotional regulation strategies, flexibility of thinking, self-concept, habituation, and revision). In addition, the participants downloaded the mobile health system ERES on their smartphones or tablets.

### Description of the ERES mobile application

**Figure 1.**
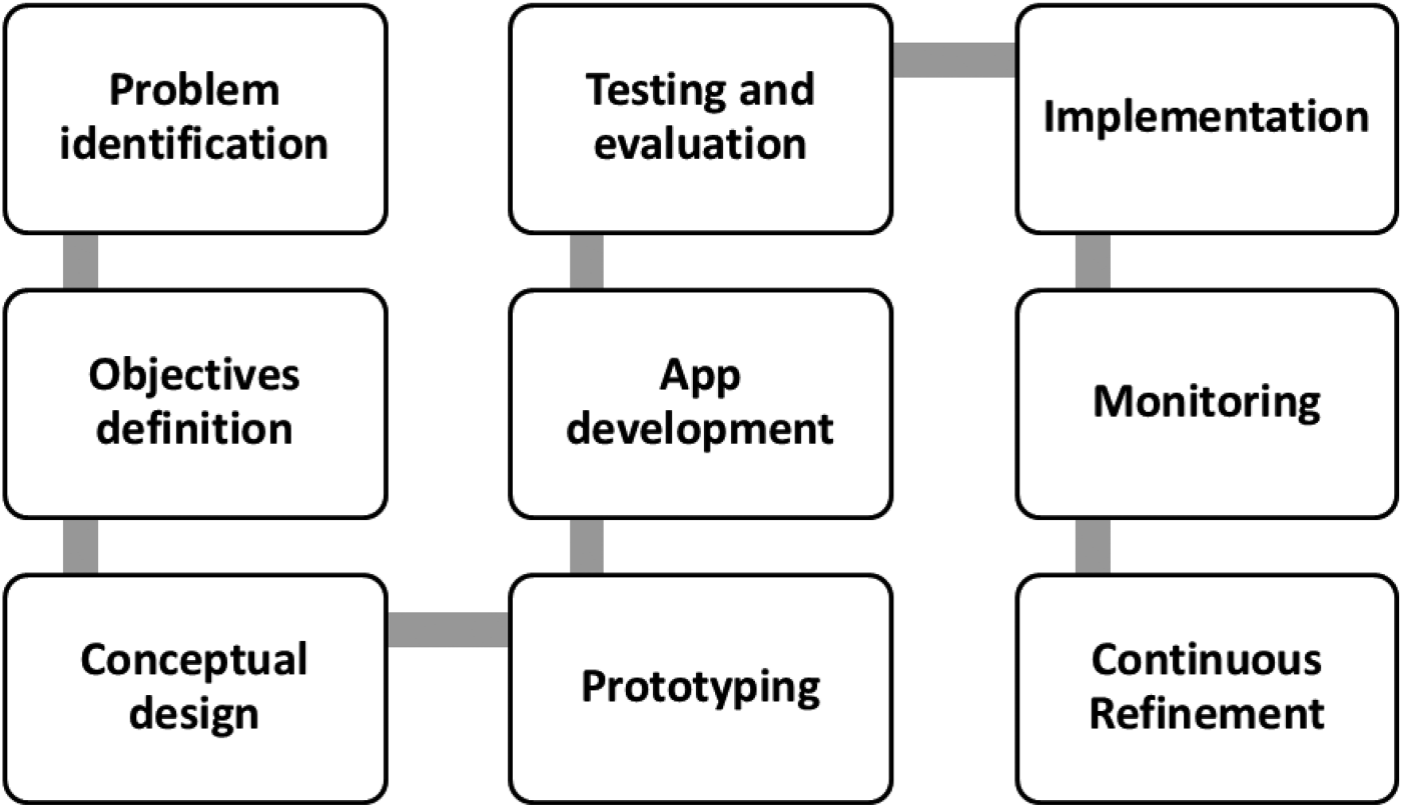
Flowchart of the ERES app design process.

The mobile app was designed and tested by a team of psychologists, educators, and software engineers who were part of the research group involved in the trial. The workflow followed for the design is schematically described in Figure 1.

It is a native mobile app for Android and IOS, which includes the following activities, adapted to the participants’ age and designed to enhance and reinforce the skills previously worked on in the face-to-face sessions:

Session 1: Developing motivation for change. Therapeutic relationship. Explanation of the app.
Session 2: What do I know about my emotions? Psychoeducation on the function of emotions and their components. The metaphor of the tornado of emotions.
Session 3. How do I deal with emotions? The metaphor of the path. The cycle of avoidance.
Session 4. How do I deal with emotions? II. Emotional regulation strategies: re-evaluation, pleasant activities, and problem-solving.
Session 5. Learning to be more flexible in our thoughts. Thought traps. Steps to get unstuck.
Session 6. Well-being in the face of uncertainty. Self-concept.
Session 7. Practicing what we have learned: I expose myself to emotional situations. Alternatives to avoidance. Habituation.
Session 8. Maintaining what we have learned. Review of emotional regulation strategies. Final summary and future.

For the users to download the app, once the consent was signed by the parents, they were provided with a QR code containing a username and password for the child or adolescent and a generic one for the parents. Once the app was downloaded and logged in, participants did not need their parents’ help to access the sessions. Parents were only responsible for ensuring that their children attended the face-to-face sessions, logged into the app, and reported any issues to us. The sessions had to follow a specific order, as the content of the online sessions is related to that of the face-to-face sessions. On a weekly basis, they were given access to the new session and received a notification that a new session was available. They could save their changes and exit the application and access as many times as necessary, as they had the entire week to complete the session. They accessed each online session sequentially. They could not start a new session early.

**Figure 2.**
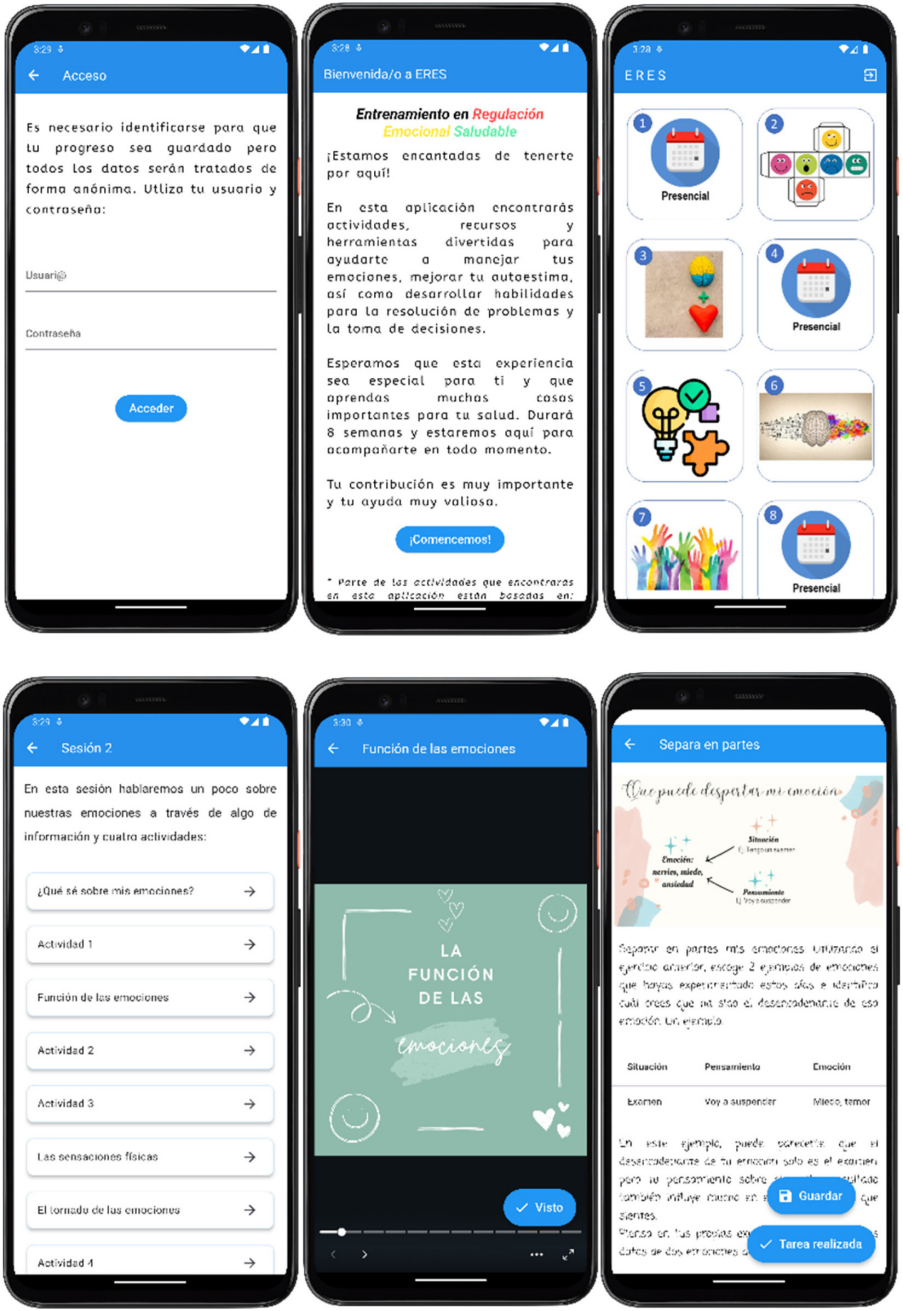
Screenshots of the mobile app ERES.

The group therapist, through a list of assigned participants, supervises the completion of activities and accesses the weekly activity program for a session (Figure 2).

### Measuring feasibility

To evaluate the app’s feasibility, we conducted a comprehensive assessment focusing on four key dimensions: adoption, usage, attrition, and usability. The percentage of participants who completed the initial pre-treatment evaluation, indicated their desire to use the mobile app, and consented to participate in the study is known as the adoption rate. The percentage of participants who download and use the program throughout the intervention period is used to determine its usage. The frequency with which each participant utilizes the app provides this information.^66^ This information has been obtained as the number of times users have accessed each session has been recorded. In the meantime, the number of participants who downloaded the app but stopped using it and did not use it again throughout the evaluation period is used to calculate the participant attrition percentage.^67^

The SUS^68^ is a 10-item scale ranging from 1 (strongly disagree, scored 0) to 5 (strongly agree, scored 4), and it is intended to evaluate the app usefulness and acceptability exclusively at the post-point. The odd-numbered items are positively worded, while the even-numbered items are negatively worded, thus balancing response biases. Once the negatively worded items are reversed, the overall score, which ranges from 0 to 100, is determined by multiplying the sum of the answers to all the items by 2.5. A score from 68 to 84 indicates good use (B rating), while a score of 85 or higher indicates exceptional usability (A rating). Additionally, this scale can offer learnability and usability subscales. These two variables are obtained by adding the scores of certain items and multiplying them by a specific number so that the score is obtained out of 100. Usability is obtained from items 1, 2, 3, 5, 6, 7, 8 and 9 and learnability from items 4 and 10.^52^ This tool has shown sufficient internal consistency in the Spanish language.^52,69^ However, to ensure understanding of the items, a version for 7–11-year-old children was double-back translated into Spanish by an expert panel.^70^ Although the children version has 13 items, only the first 10 items were used to maintain correspondence with the original version and the scoring procedure and in accordance with other studies applying this tool.^71,72^ In this study, Cronbach’s alpha for the 10-item children’s version was .81.

The ERES mobile app will be considered viable for use in minors with T1D if its adoption is similar to another previous study in which 78% is considered a high rate.^73^ A mean score above 68 on the SUS, according to Lewis and Sauro,^74^ indicates that the evaluated system has a good usability.^38,75^ This structured approach ensured a holistic understanding of the app’s practicality and its potential for sustained implementation.

### Procedure

The objective was to conduct an observational pilot study. Participants were recruited when they attended their appointments at the Endocrinology Unit of the Reina Sofía University Hospital of Córdoba (Spain). Patients who met the inclusion criteria were asked to enter the study. Parents’ participants had to sign an informed consent form designed to safeguard the confidentiality of the results obtained and to inform them of their right to leave the study at any moment without the need to provide a reason or explanation for doing so. At the beginning of the first group session, participants were gathered in a reserved room located in the same building as the hospital and were required to complete the pre-treatment assessment. They were provided with the code and invited, along with parents, to download the app on their mobile phones with the assistance of the software engineer responsible for developing the app for our study. At that point, participants received initial instructions on how to use the app correctly. Using a mobile phone with the downloaded app as a reference, they are told what they will find there, and the structure is explained to them, which is very intuitive. They are informed that each week they will receive a notification announcing that they will be able to access the corresponding session. If it is not possible to download it at that moment, they will be able to do so later. In addition, brief instructions on how to use the application appear on the initial screen. Following the download of the ERES mobile app, the group commenced its intervention sessions scheduled to take place over an 8-week period. During the final session, participants completed the post-intervention evaluation, which included the SUS. The participation runs from May 2023 to May 2024.

### Statistical analysis

For the adoption, usage, attrition, and SUS scores of mobile apps, descriptive statistics were acquired. The software developers’ team, responsible for app development, requested access to the login credentials from the ERES mobile app. IBM SPSS statistics for Windows, version 27,^76^ was used to analyze the data.

## Results

A consent form was sent to 37 patients who satisfied the inclusion criteria for the study. Twenty-eight individuals, between 7 and 18 years old, started the intervention (face-to-face + ERES app) and completed the initial assessment, establishing an adoption rate of 75.68%. Reasons for the refusal to participate in the study were as follows: transport difficulties (*n* = 3), school-related reasons (*n* = 3), and personal reasons (*n* = 3).

The use rate, defined as the proportion of participants who downloaded and accessed the application during the intervention time, was determined to be 67.86% after 19 participants (*Mean_age_* = 11.63; *SD_age_* = 2.59; 12 girls) finished the intervention and the final assessment. Nine participants withdrew from the study before it was finished, citing non-use of the app (*n* = 5), transport difficulties (*n* = 3), and issues with school (*n* = 1). Table 1 displays the participant’s characteristics.

**Table 1.** Sample characteristics (*N* = 19).

Variables		*N* (%)
Sex	Female	12 (63.2)
	Male	7 (36.8)
Age		11.63 (59)
Debut age		8.47 (2.89)
Parents’ age		41.63 (6.88)
Insulin administration	Insulin injector	17 (89.5)
	Insulin pump	2 (10.5)
N° of family members	2	1 (5.3)
	3	5 (26.3)
	4	11 (57.9)
	5	1 (5.3)
	6	1 (5.3)
N° of children with T1D	1	18 (94.7)
	2	1 (5.3)

In addition to the sociodemographic characteristics of the minors, some data on the parents were asked: most were married or in a couple and 21% divorced. Regarding the parents’ level of education, 42% said they had primary schooling, followed by 32% who answered high school level. Some 16% and 11% said they had vocational training and secondary education. Most of the parents (53%) said they had a full-time job, while 26% worked part-time. The rest were unemployed or home caregivers. In socioeconomic status, there is more variety: 42% have incomes between 10,900 and 22,000, 21% below, and 21% above. Sixteen percent said they had incomes above these figures.

The mean number of logins for all participants who completed the intervention was 8.21 (*SD* = 7.22). The following were the participants’ preferred activities based on the data they were able to access: Session 1 (*M* = 1.05, *SD* = 0.78), Session 2 (*M* = 2.16, *SD* = 1.83), Session 3 (*M* = 1.31, *SD* = 1.25), Session 4 (*M* = 0.68, *SD* = 0.58), Session 5 (*M* = 1.16, *SD* = 1.57), Session 6 (*M* = 1.10, *SD* = 1.66), Session 7 (*M* = 0.84, *SD* = 1.07), Session 8 (*M* = 0.74, *SD* = 0.87), and Resources (*M* = 1.26, *SD* = 1.41). The session that pleased participants the most was number 2. This is the first online session and consists of four sections on emotional processes, each with a title. These are: *What do I know about my emotions? The function of emotions*, *Physical sensations*, and *The emotional tornado*. In addition, there are four further sections designed to facilitate the practical application of the learning outcomes.

The mean global SUS score was 72.5 (*SD* = 14.91), which involves a “B” rating. In terms of the subscales, usability reached a score of 71.71 (*SD* = 14.37), and learnability a score of 75.66 (*SD* = 23.74). Satisfaction ratings were moderate–high for individual positive items (see Table 2). Almost all participants (52.6–94.8%) at least “somewhat agreed” with the odd items that rated the app positively, such as the one expressing their willingness to use the system frequently, or the idea that people can easily learn how to use it. Similarly, a proportion of the participants (5.3–31.6%) at least “somewhat agreed” on the even items that rated the app negatively, such as the one referring the need of the support of a technical person or the complexity of the system.

**Table 2.** Program satisfaction rating according to SUS scores (*N* = 19).

Item	*M* (*SD*)	% ≥ 4 “somewhat agree”
If I had ERES installed, I think that I would like to play it a lot	3.58 (0.77)	52.6%
I was confused many times when I was playing ERES	2.68 (1.20)	31.6%
I thought ERES was easy to use	4.53 (0.77)	94.8%
I would need help from an adult to continue to play ERES	1.74 (0.93)	5.3%
I always felt like I knew what to do next when I played ERES	3.53 (0.96)	57.9%
Some of the things I had to do when playing ERES did not make sense to me	2.05 (1.02)	5.3%
I think most of my friends could learn to play ERES very quickly	4.26 (0.80)	79%
Some of the things I had to do to play ERES were kind of weird	2.47 (1.22)	21.1%
I was confident when I was playing ERES	4.26 (0.73)	84.2%
I had to learn a lot of things before playing ERES well	2.21 (1.13)	15.8%

*Note:* Satisfaction items were rated on a 5-point scale from 1 = strongly disagree to 5 = strongly agree.

## Discussions

The apps whose feasibility has been assessed in most studies involving populations with T1D primarily consisted of continuous glucose monitoring tools. In this context, a pilot study was conducted to investigate the relationship between usage and adherence through a web-based educational training program. With a sample size of 10 and a usage rate of 75%, the study concluded that it demonstrated the ability to achieve a low attrition rate.^77^

However, other apps with a psychoeducational profile, similar to ERES—such as Teen Cope and Managing Diabetes—reported high engagement, with an adoption rate of 78% and a usage rate of 90%, in addition to a low attrition rate of 28% over 12 months.^73^ In a second study evaluating two additional apps following a similar approach—Teens.Connect and Planet D—findings indicated an adoption rate of 73%, a usage rate of 31%, and an attrition rate of 12% over 6 months.^26^

The SUS has also been employed in studies similar to this one, aimed at evaluating the usability of psychoeducational apps designed for adolescents with T1D. Resulting scores are generally very positive, ranging from 73 points in the combined use of the Diamob and Diabetes Message System apps, to 86.25 points in the Diabetes Journey app.^38,59^

The present study on the feasibility and usability of a combined intervention (face-to-face + ERES app) in a sample of pediatric patients with T1D from the Reina Sofía University Hospital in Córdoba (Spain), meeting the inclusion criteria, reported an adoption rate of 75.68%. This result reflects a high initial acceptance among participants, which could indicate strong interest from families in having their children take part in the intervention. However, the dropout rate of 32.14% suggests that almost one-third of the patients did not complete the study. The main reasons for refusal to participate were logistical difficulties, such as transportation issues (*n* = 3) and personal or school-related reasons (*n* = 3), highlighting external barriers that influenced intervention adherence. To address these limitations in future studies, potential solutions could include providing attendance certificates, conducting sessions in more convenient locations, or offering flexible scheduling, all of which could facilitate participation and reduce dropout rates.

Regarding app usage, 67.86% of participants who completed the intervention downloaded and accessed the app, indicating a moderate level of engagement. However, the attrition (32.14%), one of the primary reasons for dropout, suggests that some participants did not find the app sufficiently engaging or useful for continued use. This limitation could stem from a lack of motivation or perceived technical difficulties. To enhance adherence in future studies, modifications to the user interface, as well as the inclusion of incentives or more effective follow-up strategies, may help promote sustained app engagement. The app session most visited by participants was Session 2, which contained information about emotions in general and activities. This session is the first contact with the app.

Participant satisfaction, assessed using the SUS, showed an overall score of 72.5 (B rating), indicating that users generally found the app easy to use and learn. Subscale scores for usability (71.71) and learnability (75.66) further support this positive evaluation. However, some participants expressed a need for technical support and perceived the app as complex, as reflected in responses to negatively framed questionnaire items, where a smaller proportion of participants (5.3–31.6%) agreed with these concerns. To mitigate these issues, providing guidance during the first app access, offering additional tutorials, or ensuring readily available technical support could enhance the user experience.

Despite the high overall satisfaction, logistical challenges and difficulties related to motivation for continued app use emphasize the need for refinements in the intervention. Dropout rates could be reduced by improving app accessibility and perceived relevance, as well as implementing measures to support participants throughout the process. Additionally, greater flexibility in intervention delivery, tailored to the needs and schedules of participants, could help maximize adherence.

In summary, the results suggest that the intervention is feasible and usable, but key areas for improvement include increasing app engagement and participant retention. Addressing logistical and motivational barriers and optimizing the user experience are crucial steps toward enhancing intervention effectiveness in future research.

When comparing the data with similar studies’ findings, we observe that ERES achieves an initiation rate within the average range; however, a significant number of participants do not continue. In this case, for the future main intervention, we could focus on understanding the reasons for attrition in order to mitigate them and enhance these figures. Additionally, we could pay attention to the lowest-rated items of the SUS to optimize ERES.

These findings underscore the importance of developing engaging and accessible digital interventions for children and adolescents with T1D. By improving usability and addressing adherence barriers, future research can enhance the effectiveness of psychological support tools, ultimately improving the quality of life of this population. Continued investigation in this field holds the potential to develop impactful solutions that promote better health outcomes and empower young patients in their self-management journey

Despite advances, research on psychological interventions for children with T1D remains limited in scope and quantity. This gap in knowledge emphasizes the need for greater attention and resources for research that specifically approaches the unique needs and challenges faced by these children and their families.^78^ In response to this need, online psychological therapies are being developed to improve the emotional well-being of these individuals, thus providing them with a crucial opportunity for support and improvement in their quality of life.^26^ In parallel, eHealth interventions have great impact on public health.^79^ The increasing use of the Internet and mobile devices worldwide has made these interventions increasingly common.^49^ However, the scientific evidence in this area is very limited and recent. More studies integrating these treatments into clinical practice, as well as economic analyses of this type of intervention versus the traditional face-to-face model, are needed in order to make solid conclusions. Moreover, this is such a novel topic that it is difficult to compare our application with other previous ones with the same characteristics to measure its quality. However, as the aim of the study was to evaluate the feasibility of the app in a sample of children and adolescents with T1D, the adoption, usage, and usability data suggest that this app could potentially be used in larger trials with a larger patient population and longer timings.

## Limitations

The main limitation of this study was the dropout of a significant part of the sample. As this is an important variable for evaluating treatment, we have registered the reasons for dropping out. In this case, the causes are related to issues with school, difficulties in attending, and non-use of the app. In the review by Melville et al.,^80^ dropout in online mental health treatment programs was studied, in which a mean dropout rate of 31% was obtained; however, there was no evidence of any specific variable predicting dropout. The follow-up meta-analysis by de Haan et al.^81^ in a child population concluded that there were more predictors of dropout related to treatment, therapist, or participation than to children and their families. This is, therefore, a key as to where we should pay attention when it should be possible to reduce the percentage of dropouts in future experiments.

## Conclusions

We present an app that is part of a psychological intervention for children with T1D, which showed an attrition of 32.14%, mainly due to lack of app use and logistical barriers. To improve adherence, future interventions should enhance accessibility, motivation strategies, and technical support. However, the app was well rated by users (SUS = 72.5, “B” rating), with high scores in usability (71.71) and learnability (75.66), indicating that it is intuitive and easy to use. These results highlight its potential as a supportive tool in psychological interventions for this population.
